# Improvement in Quality Metrics Outcomes and Patient and Family Satisfaction in a Neurosciences Intensive Care Unit after Creation of a Dedicated Neurocritical Care Team

**DOI:** 10.1155/2017/6394105

**Published:** 2017-10-08

**Authors:** Yaw Sarpong, Premkumar Nattanmai, Ginger Schelp, Robert Bell, Keerthivaas Premkumar, Erin Stapleton, Ashley McCormick, Christopher R. Newey

**Affiliations:** ^1^Department of Surgery, Division of Neurosurgery, University of Missouri, 1 Hospital Drive, Columbia, MO 65211, USA; ^2^Department of Neurology, University of Missouri, 5 Hospital Drive, CE 540, Columbia, MO 65211, USA; ^3^University of Missouri Health System, 1 Hospital Drive, Columbia, MO 65211, USA; ^4^Department of Pharmacy, University of Missouri, 1 Hospital Drive, Columbia, MO 65211, USA

## Abstract

**Introduction:**

Dedicated neurointensivists have been shown to improve outcome measurements in the neurosciences intensive care unit (NSICU). Quality outcome data in relation to patient and family satisfaction is lacking. This study evaluated the impact of newly appointed neurointensivists and creation of a neurocritical care team on quality outcome measures including patient satisfaction in a NSICU.

**Methods:**

This is a retrospective study of data over 36 months from a 14-bed NSICU evaluating quality outcome measures and anonymous patient satisfaction questionnaires before and after neurointensivists appointment.

**Results:**

After appointment of neurointensivists, patient acuity of the NSICU increased by 33.4% while LOS decreased by 3.5%. There was a decrease in neurosciences mortality (35.8%), catheter-associated urinary tract infection (50%), central line associated bloodstream infection (100%), and ventilator-associated pneumonia (50%). During the same time, patient satisfaction increased by 28.3% on physicians/nurses consistency (*p* = 0.025), by 69.5% in confidence/trust in physicians (*p* < 0.0001), by 78.3% on physicians treated me with courtesy/respect (*p* < 0.0001), and by 46.4% on physicians' attentiveness (*p* < 0.0001). Ultimately, patients recommending the hospital to others increased by 67.5% (*p* < 0.0001).

**Conclusion:**

Dedicated neurointensivists and the subsequent development of a neurocritical care team positively impacted quality outcome metrics, particularly significantly improving patient satisfaction.

## 1. Introduction

Neurointensive care is an area of medicine bridging multiple fields to provide specialized care to critically ill patients with neurological illnesses [1, 2]. The neurointensivist serves an important role orchestrating personnel, neurologists, neurosurgeons, consultants, therapists, pharmacists, nursing, and administration, involved in patient care in the neurosciences intensive care unit (NSICU). This orchestrater role is important for patient outcome and healthcare quality [3, 4]. Studies have shown that a neurointensivist managing a NSICU improves outcomes and shortens length of stays in all neurocritically ill patients and also improves documentation [5–9]. Similar improvements after dedicated neurointensivists have been demonstrated in patients with ischemic stroke [9–14], subarachnoid hemorrhage [15, 16], traumatic brain injury [17, 18], intracerebral hemorrhage [19], and neuromuscular respiratory failure [20]. These studies, however, lack information on patient and family satisfaction concomitantly with the improvement in other clinical quality metrics.

The purpose of this study was to evaluate the impact of newly appointed neurointensivists with the subsequent development of a neurocritical care team on quality metrics including patient and family satisfaction after discharge from a NSICU at an academic, tertiary care medical center.

## 2. Methods

This was a retrospective study of anonymous patient surveys and quality metric data from a 14-bed NSICU at an academic, tertiary care center over a 36-month time period (January 2014 to January 2017). 18 months were reviewed before appointment of neurointensivists and were compared to a similar 18-month time frame after appointment of neurointensivists (CRN and PN). The Institutional Review Board approved this study.

### 2.1. Survey Patient Population

Quality metrics data included catheter-associated urinary tract infection (CAUTI), Foley catheter days, central line associated blood stream infection (CLABSI), central line days, ventilator-associated pneumonia (VAP), ventilator days, patient acuity, mortality (observed : expected ratio), length of stay (LOS), and patient days. These metrics were obtained from University Health System Consortium (UHC; Irving, TX, USA), National Healthcare Safety Network (NHSN; Atlanta, GA, USA), and Acuity Plus databases (Herndon, VA, USA). Anonymous survey data was obtained from patient satisfaction questionnaires after discharge from the NSICU using the National Research Council (NRC) Picker database (Lincoln, NE, USA). The Likert scale questionnaire included questions related to (1) physicians and nurses consistency; (2) confidence and trust in physicians; (3) courtesy and respect by physicians; (4) attentiveness of physicians; (5) recommending the hospital to others. The questions can be found in Table 1. Data were compared after the appointment of neurointensivists (CRN and PN) and dedicated neurocritical care team to controls from the immediate 18 months before appointment. Before the appointment of neurointensivists, the NSICU was managed as an open ICU by neurologists and neurosurgeons with the assistance of consulting medical intensivists and advanced practice providers. Not all patients required a consultation from the medical intensive care team. After the appointment of the neurointensivists and creation of the neurocritical care team, the NSICU operated as a high intensity ICU model.

### 2.2. Establishment of the NSICU

#### 2.2.1. Clinical Improvement

The main focus was on clinical improvement in key areas: ICU team, coordination of care, family and patient involvement in their care, nursing involvement during rounding, and creation of checklists, protocols, and order sets. The NSICU team worked closely with the admitting service, neurology or neurosurgery, to develop thorough plans for the patients' care.

Rounding occurred at the bedside in patients' rooms. The neurointensivist directed the rounding. Families were encouraged to participate in rounding. Nurses provided a brief overview of the patient followed by neurological examination and neurological specific data (e.g., external ventricular device setting and output, changes in examination, planned neuroimaging, sedation, and pain control) followed by other pertinent information and data (e.g., vitals, ins and outs, bowel regimen and diet, blood glucose trends, respiratory status, inflammatory/infectious status, ending with deep venous thrombosis (DVT) prophylaxis, and skin concerns). Immediately following the nurse presentation, the dedicated neuropharmacist reviewed medications with the team. The resident and/or advanced practice providers then discussed the plan by organ system with the neurointensivist. This format allowed for bedside teaching of management of the neurocritically ill patient. The resident/advance practice providers' progress note was specific to the needs of the critically ill neurosciences patient and followed the same format as the nurses rounding report. Finally, an eleven-point checklist ensured basic care of an ICU patient was completed. This checklist was based on the “ABCDEF” ICU bundle (http://www.icudelirium.org/). The rounding NSICU team consisted of nurses, advance practice providers, residents, medical students, critical care fellows rotating from other units, pharmacist, and respiratory therapist.

Prior to formal rounding each morning, a multidisciplinary meeting with the neurology, neurosurgery, and NSICU teams occurred to discuss all patients in the NSICU. At this meeting, the plans of care were reviewed with social work, case management, nutrition, speech/language pathology, respiratory therapy, and physical and occupational therapy to ensure consistent disposition plan. A separate huddle also occurs with nursing prior to formal rounds to review the need for catheters, central lines, and ventilators.

To ensure maintenance of quality, a weekly meeting occurred with nursing leadership of the NSICU in addition to the neurosurgery and neurology service leadership to discuss nursing and unit concerns and improvement opportunities. During these meetings, we addressed mortalities, documentation, grievances, and future plans. Additional monthly meetings with infection control reviewed VAPs, CAUTIs, mortalities, and patient safety network concerns.

Protocols and order sets were created specific for the NSICU. These served as a basis for educating other members of the team as well as standardizing care delivered.

#### 2.2.2. Education and Research

Education of residents, nurses, medical students, and critical care fellows was emphasized. Weekly meetings were held to lecture on topics specific to NSICU, discuss journal articles, review mortality/morbidity, discuss research opportunities, and review electroencephalograms (EEGs) from NSICU patients.

### 2.3. Statistics

Continuous and categorical data were summarized with descriptive statistics including means and standard deviations (continuous data) and frequencies (categorical data). Fisher's exact test (categorical) and Student's *t*-test (continuous) were used for statistical analyses. A *p* ≤ 0.05 was considered significant. Data was analyzed using GraphPad Prism 7 (LaJolla, CA, USA).

## 3. Results

### 3.1. Quality Metrics

There were 4426 total patient days before appointment of dedicated neurointensivists and 4496 total patient days after appointment of dedicated neurointensivists and neurocritical care team over the 36-month study period. After the appointment, patient acuity of the NSICU increased by 3.4% (5.41 versus 7.21; *p* = 0.13) while LOS decreased by 3.5% (13.11 versus 12.66; *p* = 0.77). Neurology/neurosurgery mortality observed-to-expected ratio decreased by 35.8% (1.49 versus 0.90; *p* = 0.019). Absolute decreases were seen in CAUTI (50%, *p* = 0.41), CLABSI (100%, *p* = 0.51), and VAP (50%, *p* = 0.57). Overall, there was a significant decrease in central line days (1.5%, *p* < 0.0001), ventilator days (0.66%, *p* < 0.0001), and Foley days (18.3%, *p* < 0.0001; Table 2).

### 3.2. Patient Satisfaction

**Figure 1:**
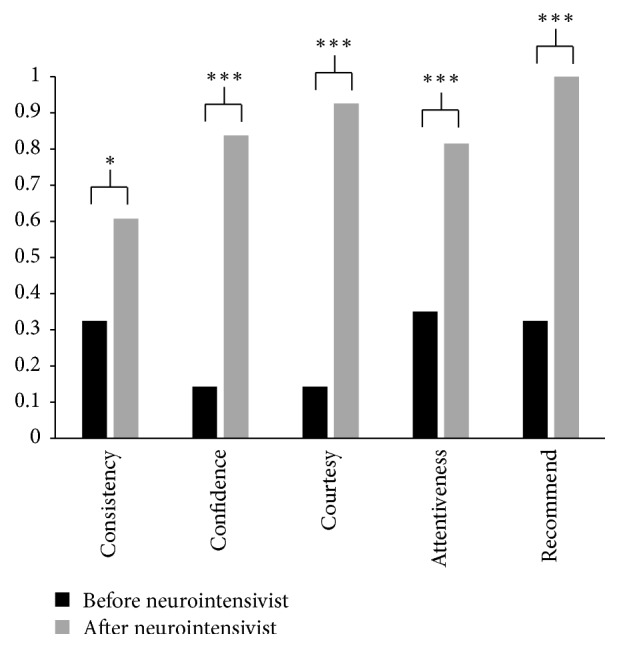
Patient Satisfaction Scores. Patient satisfaction scores from questionnaire data are illustrated. Before neurointensivists appointment is noted in black bars. After neurointensivist appointment is noted in gray bars. Significant improvements in scores were observed with all questions after neurointensivist appointment.   ^*∗*^*p* < 0.05; ^*∗∗∗*^*p* < 0.0001.

114 questionnaires were returned. Comparing the questionnaires before and after neurointensivists (*n* = 77, and *n* = 37, resp.), patient satisfaction increased by 28.3% on physicians and nurses consistency (*p* = 0.025), by 69.5% in confidence and trust in physicians (*p* < 0.0001), by 78.3% on courtesy and respect by the physicians (*p* < 0.0001), and by 46.4% on physicians' attentiveness (*p* < 0.0001). Patients recommending the hospital to others increased by 67.5% (*p* < 0.0001, Figure 1).

## 4. Discussion

Dedicated neurointensivists and the creation of a neurocritical care team positively impacted quality outcome metrics including decreasing the rates of mortality, CAUTI, CLABSI, and VAP and ultimately decreasing LOS. Importantly, the appointment of neurointensivists positively impacted patient satisfaction. This study is the first to highlight improvement in patient satisfaction in addition to quality metric data after the appointment of neurointensivists.

After the appointment of neurointensivists, the acuity of the NSICU increased. Despite this increase in acuity, the mortality and length of stay decreased. Prior to the appointment of neurointensivists, higher acuity patients were admitted to the medical intensive care unit with neurology consulting. These critically ill neurosciences patients were now managed by neurointensivists in the NSICU. We hypothesized that achieving these goals of decreasing LOS and mortality was largely in part because of having specially trained neurointensivists and dedicated neurocritical care team managing the critically ill patient, establishing protocols and order sets specific to neurologically injured patients, and focusing on nursing education and engagement. The multidisciplinary approach to the patient created an open discussion with nursing and therapists about the patients. This approach possibly improved our CAUTI, CLABSI, and VAP rates likely by the reduction in our line and catheter days. The absolute numbers are small, which may overstate the significance. Ventilator days also significantly decreased by a small absolute amount (i.e., 7 ventilator days).

The decrease in the observed-to-expected mortality ratio (i.e., a risk-adjusted measure of the overall mortality) can be accomplished by two methods. One is to increase the expected mortality. The other is to decrease the observed mortality. The expected mortality is calculated based on premorbid and comorbid conditions recorded in a patient population that are beyond control of the hospital. The observed mortality is the actual number of deaths. It can be argued that documentation improved with the appointment of the neurointensivists, which ultimately decreased the observed-to-expected ratio. However, prior to the appointment of the neurointensivists, the institution had standardized collection of key coding words in admission and progress notes for the expected mortality variable, which has largely remained unchanged. Overall, the decrease in the observed-to-expected rate was likely a combination of improved documentation (which increased the expected mortality denominator) as well as an actual decrease in mortality (which decreased the observed mortality numerator).

Our data is consistent with the retrospective study by Suarez et al. [5]. This group showed that after the introduction of a neurointensivist and neurocritical care team, mortality and length of stay significantly decreased. They hypothesized that the driving factors of the improved outcomes were from ICU organization including staffing with physicians that were readily available to critically ill patients as well as creating a high intensity intensive care unit model [5]. The benefits of a high intensity model have been shown by Durbin Jr. [3]. In a high intensity model of ICU care delivery, where all orders are placed by ICU team and all patients in the ICU are seen by the ICU team, mortality decreased (OR 0.61; 95% CI 0.05–0.75) and ICU and hospital lengths of stay decreased [3, 21]. Durbin Jr. estimated that 30–50% fewer patients would die if an intensivist rounded daily on all critically ill patients [3]. They hypothesized that this model allowed for more rapid intervention and prevention of potentially catastrophic events [3]. Indeed, in the medical ICU, mortality from septic shock decreased from 74 to 57% by having an intensivist and all ICU mortality decreased from 20.9 to 14.9% [3]. Mortality actually increased by a factor of three if there was not a daily ICU physician led round [3, 8]. Finally, the intensivist was defined as a person who “makes* all final decisions* about the care of the patients, including who is admitted and discharged, which physicians to consult, and all other aspects of care” [22].

Barriers to work as a team are known. These include physicians and the health professionals being slow to accept change, relinquishing some autonomy to become a team member, other physicians feel loss of authority, patient control, and personal income [3]. A well-functioning team has been shown to provide better care than a single physician dictating care from a remote site [3].

The benefits of a critical care team extend beyond just having a dedicated intensivist. Other factors that improve patient outcomes include keeping nurse-to-patient ratio of less than 1 : 2 [3, 22], having a dedicated pharmacist on rounds [3], and having a dedicated respiratory therapist [3]. We have also shown that the rates of CAUTI, CLABSI, and VAPs decreased after development of a dedicated team managing the critically ill neurosciences patient. The decreased infection rate from CLABSI, CAUTI, and VAP can be cost saving based on 2016 national average data provided by the Health Services Advisory Group (https://www.hsag.com/). Indeed, managing neurologically injured patients in a dedicated neurocritical care unit is cost-effective compared to combined neuro/general critical care units [21].

Patient satisfaction improved with the development of a neurocritical care team led by a neurointensivist. We have shown that patient and family satisfaction improved on physician attentiveness and consistency, confidence/trust in the physician, and treatment with respect and courtesy. Ultimately, the patients and families recommended the hospital to others. These improvements reflect the high intensity model that was created. By having an intensivist led ICU, the admitting physician (either neurology or neurosurgery) can spend more time performing surgery and/or seeing patients in clinic by being freed of the need to provide care on a minute-to-minute basis to critically ill patients [3]. This model has been shown to be less alienating compared to a closed ICU model [3]. The high intensity model encourages communication between the different services, which ultimately provides the best care for the patient. In the neurocritical care unit, this model has been shown to be effective [8, 14]. An important aspect we noticed was our rounding style. Rounding occurred at bedside, which encouraged patients and families to participate in rounds and decisions. This type of rounding style allowed for transparency in decision making. It also encouraged open communication between the families, patients, and neurocritical care team.

In comparison to the other satisfaction questions, physician and nurse consistency only improved by 28.3% with the development of the neurocritical care team. We suspect this was a reflection of a high intensity model (i.e., not a closed ICU model) as well as the inherent nature of an intensive care unit. Nurses worked three 12-hour shifts that caused constant change and less continuity with care of the patient requiring a prolonged ICU stay. Nurses also rotated from other units without having the specialized foundation of neurocritical care that is necessary to communicate with families and patients. Similarly, residents rotated on two-week blocks through the NSICU. Night-time was covered by a night-float resident who may not fully understand the details of the plan as discussed during the day. Rotating residents come from varying backgrounds - neurology, neurosurgery, emergency medicine, and family medicine. Lastly, the timing of rounds by the other services can be a source of miscommunication. The results of the patient satisfaction questionnaire and possible sources for inconsistencies have provided us with information on areas for improvement.

Improvement in patient satisfaction and quality metrics have become increasingly crucial in reimbursements from payors. The restructuring of reimbursements by health insurances places specific emphasis on quality metrics and patient satisfaction [23]. Future projects will be aimed at improving consistency in the communication with patients and families and ultimately determining the financial benefits to the hospital by the improved patient satisfaction scores.

Retrospective studies have inherent limitations. A limitation of this study was that not all families/patients returned questionnaires or fully completed questionnaires. It is unclear how many questionnaires were originally mailed to patients. The questionnaires were administered by an independent agency. Thus, we cannot calculate the response rate. The questionnaires were answered after discharge from hospital; thus, answers may be biased by post-ICU care. We limited our analysis to the specific questions relating to the NSICU. We do recognize that the care patients receive after ICU can bias their overall reflection of the hospital experience. Additionally, the number of surveys returned after the appointment of the neurointensivists was less than before appointment. This discrepancy was a reflection of the growth of the ICU. The acuity of the patients in the ICU has increased since the neurointensivists were appointed. As such, many of these patients after neurointensivist appointment were discharged to a long-term acute care facility, skilled nursing facility, or acute rehabilitation center. Patients who were not discharged home were not surveyed. Lastly, we do not have data from other intensive care units during this same time period for comparison.

To overcome the limitations, families were actively engaged in rounds. A nursing position was created to focus on education and research. The neurocritical care team worked with nursing and neurology/neurosurgery leadership on methods to improve consistent message to the patients/families. The messages emphasized included team discussions prior to prognosticating, open communication, and increased interface with the other services, simplifying the clinical information provided to families/patients and improving the structure of nursing and multidisciplinary huddles.

In conclusion, our data is the first to show improvement in anonymous satisfaction scores from patients discharged from a NSICU in relation to other quality metrics after the implementation of a neurointensivist-led, high intensity ICU model. Our data is consistent with prior studies showing improvement in iatrogenic infections and a decrease in mortality and LOS with dedicated neurointensivists and a neurocritical care team.

## Figures and Tables

**Table 1:** Patient satisfaction questionnaire.

Questions	
(1)	How often were the different doctors and nurses consistent with each other in providing you information and care?
(2)	During this hospital stay, how often did you have confidence and trust in the doctors treating you?
(3)	During this hospital stay, how often did doctors treat you with courtesy and respect?
(4)	During this hospital stay, how often did doctors listen carefully to you?
(5)	Would you recommend this hospital to your friends and family?

**Table 2:** Quality metric data.

	Before neurointensivist		After neurointensivist		*p* value
Total patient (days)	4426		4496		
Acuity (*n*, SD)	5.41	2.38	7.21	3.76	0.13
Mortality O : E (*n*, SD)	1.49	0.48	0.9	0.51	**0.019**
LOS (days, SD)	13.11	4.7	12.66	4.17	0.77
Foley (days, SD)	3097	17.63	2323	20.01	**<0.0001**
CAUTI (*n*, %)	16	0.52	8	0.34	0.41
Central line (days, SD)	1598	25.79	1557	27.12	**<0.0001**
CLABSI (*n*, %)	2	0.13	0	0	0.51
Ventilator (days, SD)	1429	22.02	1422	22.71	**<0.0001**
VAP (*n*, %)	2	0.14	1	0.07	0.57

*n*: number; SD: standard deviation; O : E: observed : expected ratio; LOS: length of stay; CAUTI: catheter-associated urinary tract infection; CLABSI: central line associated bloodstream infection; VAP: ventilator associated pneumonia.

## References

[1] Wijdicks E. F. M., Worden W. R., Miers A. G., Piepgras D. G. (2011). The early days of the neurosciences intensive care unit. *Mayo Clinic Proceedings*.

[2] Ward M. J., Shutter L. A., Branas C. C., Adeoye O., Albright K. C., Carr B. G. (2012). Geographic access to US neurocritical care units registered with the neurocritical care society. *Neurocritical Care*.

[3] Durbin C. G. (2006). Team model: Advocating for the optimal method of care delivery in the intensive care unit. *Critical Care Medicine*.

[4] Mirski M. A., Chang C. W. J., Cowan R. (2001). Impact of a neuroscience intensive care unit on neurosurgical patient outcomes and cost of care: Evidence-based support for an intensivist-directed specialty ICU model of care. *Journal of Neurosurgical Anesthesiology*.

[5] Suarez J. I., Zaidat O. O., Suri M. F. (2004). Length of stay and mortality in neurocritically ill patients: impact of a specialized neurocritical care team. *Critical Care Medicine*.

[6] Sarpong Y., Nattanmai P., Schelp G. Importance of neurocritical care team in patient and family satisfaction in a neuroICU.

[7] Sarpong Y., Nattanmai P., Schelp G. Importance of neurocritical care team in patient and family satisfaction in a neuroICU.

[8] Varelas P. N., Conti M. M., Spanaki M. V. (2004). The impact of a neurointensivist-led team on a semiclosed neurosciences intensive care unit. *Critical Care Medicine*.

[9] Varelas P. N., Spanaki M. V., Hacein-Bey L. (2005). Documentation in medical records improves after a neurointensivist's appointment. *Neurocritical Care*.

[10] Langhorne P. (1997). Collaborative systematic review of the randomised trials of organised inpatient (stroke unit) care after stroke. *British Medical Journal*.

[11] Dayno J. M., Mansbach H. H. (1999). Acute stroke units. *Journal of Stroke and Cerebrovascular Diseases*.

[12] Group TEAHC (2004). Optimizing Intensive Care in Stroke: A European Perspective. *Cerebrovascular Diseases*.

[13] Gujjar A. R., Deibert E., Manno E. M., Duff S., Diringer M. N. (1998). Mechanical ventilation for ischemic stroke and intracerebral hemorrhage: Indications, timing, and outcome. *Neurology*.

[14] Varelas P. N., Schultz L., Conti M., Spanaki M., Genarrelli T., Hacein-Bey L. (2008). The impact of a neuro-intensivist on patients with stroke admitted to a neurosciences intensive care unit. *Neurocritical Care*.

[15] Enblad P., Persson L. (1997). Impact on clinical outcome of secondary brain insults during the neurointensive care of patients with subarachnoid haemorrhage: A pilot study. *Journal of Neurology Neurosurgery and Psychiatry*.

[16] Samuels O., Webb A., Culler S., Martin K., Barrow D. (2011). Impact of a dedicated neurocritical care team in treating patients with aneurysmal subarachnoid hemorrhage. *Neurocritical Care*.

[17] Elf K., Nilsson P., Enblad P. (2002). Outcome after traumatic brain injury improved by an organized secondary insult program and standardized neurointensive care. *Critical Care Medicine*.

[18] Varelas P. N., Eastwood D., Yun H. J. (2006). Impact of a neurointensivist on outcomes in patients with head trauma treated in a neurosciences intensive care unit. *Journal of Neurosurgery*.

[19] Burns J. D., Green D. M., Lau H. (2013). The effect of a neurocritical care service without a dedicated neuro-ICU on quality of care in intracerebral hemorrhage. *Neurocritical Care*.

[20] Varelas P. N., Chua H. C., Natterman J. (2002). Ventilatory care in myasthenia gravis crisis: assessing the baseline adverse event rate. *Critical Care Medicine*.

[21] Harrison D. A., Prabhu G., Grieve R. (2013). Risk Adjustment In Neurocritical care (RAIN) - prospective validation of risk prediction models for adult patients with acute traumatic brain injury to use to evaluate the optimum location and comparative costs of neurocritical care: A cohort study. *Health Technology Assessment*.

[22] Gutsche J. T., Kohl B. A. (2007). Who should care for intensive care unit patients?. *Critical Care Medicine*.

[23] Taylor-Rick L. Patient satisfaction plays role in medicare reimbursement.

